# Emotion Regulation in Rescue Workers: Differential Relationship With Perceived Work-Related Stress and Stress-Related Symptoms

**DOI:** 10.3389/fpsyg.2018.02744

**Published:** 2019-01-10

**Authors:** Anne Gärtner, Alexander Behnke, Daniela Conrad, Iris-Tatjana Kolassa, Roberto Rojas

**Affiliations:** ^1^Differential and Personality Psychology, Faculty of Psychology, Technische Universität Dresden, Dresden, Germany; ^2^Clinical and Biological Psychology, Institute of Psychology and Education, Ulm University, Ulm, Germany; ^3^Clinical and Neuropsychology, Department of Psychology, University of Konstanz, Konstanz, Germany; ^4^Universitary Psychotherapeutic Outpatient Clinic, Institute of Psychology and Education, Ulm University, Ulm, Germany

**Keywords:** rescue workers, emergency medical technicians, emotion regulation, rumination, post-traumatic symptoms, depressive symptoms

## Abstract

Rescue workers are exposed to enduring emotional distress, as they are confronted with (potentially) traumatic mission events and chronic work-related stress. Thus, regulating negative emotions seems to be crucial to withstand the work-related strain. This cross-sectional study investigated the influence of six emotion regulation strategies (i.e., rumination, suppression, avoidance, reappraisal, acceptance, and problem solving) on perceived work-related stress and stress-related depressive, post-traumatic, and somatic symptoms in a representative sample of 102 German rescue workers. Multiple regression analyses identified rumination and suppression to be associated with more work-related stress and stress-related symptoms. Acceptance was linked to fewer symptoms and, rather unexpectedly, avoidance was linked to less work-related stress. No effects were observed for reappraisal and problem solving. Our findings confirm the dysfunctional role of rumination and suppression for the mental and physical health of high-risk populations and advance the debate on the context-specific efficacy of emotion regulation strategies.

## Introduction

Rescue workers are required to respond to a variety of emergency situations involving human suffering, danger, and death. For example, their occupational work includes providing emergency medical assistance to injured people and rescuing humans from accidents, fires, floods, or other natural or human-made disasters. Consequently, rescue workers are regularly confronted with traumatic events (e.g., Regehr et al., 2002; Marmar, 2006). That is, they are confronted (directly or witnessing) with actual or threatened death, serious injury, sexual violence, and/or serious aversive details of those events. These situations go along with physical, psychological, and emotional stress. Subsequently, rescue workers are at higher risk for experiencing strong negative emotions (e.g., fear, worry), and disturbed sleep or concentration (e.g., Van Der Ploeg and Kleber, 2003; Benedek et al., 2007; Halpern et al., 2009; Donnelly et al., 2016), which in turn promotes the development of physical and mental health problems. Indeed, the more often rescue workers are confronted with traumatic events on duty, the higher is their risk of clinically significant and often comorbid depressive and post-traumatic symptoms as well as physical complaints (Teegen and Yasui, 2000; Fullerton et al., 2004; Benedek et al., 2007; Berger et al., 2012; Donnelly, 2012; Razik et al., 2013; Fjeldheim et al., 2014; Wild et al., 2016; Skeffington et al., 2017). In addition to their duty-related trauma exposure, rescue workers’ individual vulnerability for mental health problems may further be increased by nonwork-related traumatic events in private life, particularly experiences of childhood maltreatment (Maunder et al., 2012; for reviews, see Hamilton et al., 2015; Li et al., 2016).

Besides the experience of traumatic events, the burden of rescue workers is further complicated by their workload, resulting from adverse working conditions such as shiftwork and its known negative consequences on physical and mental health (for a review see, Frank and Ovens, 2002). Further factors are chronic workplace stress such as false alarms, time pressure and tensed relationships with colleagues and managers due to increased stress in an already high-stress profession (Clohessy and Ehlers, 1999; Teegen and Yasui, 2000; Van Der Ploeg and Kleber, 2003; Aasa et al., 2005; Heringshausen et al., 2010). From a more general point of view, the economic situation also impacts on worker’s health in non-profit organizations such as the rescue service. In detail, studies have shown that major macroeconomic distortions (such as the economic crisis) have a negative impact on health care services due to decreased private and public funding, resulting in increased workload, job insecurity, reduction of wages, and fear of becoming unemployed (Giorgi et al., 2015; Mucci et al., 2016). Indeed, the economic recession has been linked to the development of mental illness due to increased work-related stress (reviewed in Mucci et al., 2016). In this regard, the economic recession could contribute to an increased stress load together with already unfavorable working conditions and the experience of traumatic events in rescue workers. Interestingly, accumulated stress has been associated with chronic low-grade inflammation which in turn represents an underlying biological mechanism in the development of mental disorders such as depression and PTSD (Berk et al., 2013; Gola et al., 2013; Boeck et al., 2016). In sum, several factors might lead to the perception of increased work-related stress in rescue workers, which accelerates the development of post-traumatic, depressive, and somatic symptoms (Beaton et al., 1997; Boudreaux et al., 1997; Aasa et al., 2005; Bennett et al., 2005; Sterud et al., 2008; Wild et al., 2016). These mental and physical health problems can in turn cause sickness-related absence from work, earlier retirement and even complete withdraw from the job (Hammer et al., 1986; Dirkzwager, 2004; Chapman et al., 2009; Razik et al., 2013).

Given the work-related stressors and constant exposure to potential traumatic events on duty, the ability to deal with negative emotions seems to constitute a crucial component in the daily duties of rescue workers to stay healthy and carry out their work properly. Emotion regulation is defined as all processes by which individuals influence which emotions they have, when they have them, and how they experience and express them (Gross, 1998, 2015). While several emotion regulation strategies have been associated with personal well-being, physical and mental health (“adaptive” strategies), others have been proposed to boost the vulnerability to develop mental problems (“maladaptive” strategies; for reviews see, Aldao and Nolen-Hoeksema, 2010; Aldao et al., 2010). To the first category belongs the attempt to change a stressful or negative situation and its consequences (*problem solving*); generating positive interpretations or perspectives on a negative situation in order to reduce negative emotions (*reappraisal*); and the ability to accept emotions, thoughts, and perceptions without evaluating them (*acceptance*; Gross, 1998; Aldao et al., 2010). In contrast, typical maladaptive emotion regulation strategies are the tendency to repetitively focus on the experience of negative emotions and its causes and consequences (*rumination*); the suppression of the emotional expression and experience of negative emotions (*suppression*); and the avoidance of thoughts, emotions, sensations and memories related to the negative event (*avoidance*; Foa and Kozak, 1986; Wenzlaff and Wegner, 2000; Gross and John, 2003; Garnefski and Kraaij, 2006; Nolen-Hoeksema et al., 2008).

These strategies are commonly studied in the context of psychopathology in healthy and clinical populations, where research identified moderate to large effect sizes. In detail, studies have shown that the use of adaptive emotion regulation strategies (particularly reappraisal and problem solving) is linked to enhanced resilience against negative emotional stress and mental diseases (for meta-analyses see Aldao and Nolen-Hoeksema, 2010; Aldao et al., 2010; Webb et al., 2012). In addition, reappraisal and acceptance were found to promote post-traumatic growth (Prati and Pietrantoni, 2009). On the contrary, the use of maladaptive strategies was consistently associated with increased negative emotional stress and elevated risk for mental health problems (for meta-analyses see Aldao and Nolen-Hoeksema, 2010; Aldao et al., 2010; Webb et al., 2012).

Research on maladaptive emotion regulation in trauma-exposed individuals such as rescue workers yielded relatively consistent results. Previous research found rumination, suppression, and avoidance to be associated with more severe post-traumatic and depressive symptoms (e.g., Beaton et al., 1999; Clohessy and Ehlers, 1999; Kirby et al., 2011; Razik et al., 2013; Shepherd and Wild, 2014; Wild et al., 2016). More important, longitudinal studies indicated that rumination represents a prospective risk factor for the development of PTSD and depression (Wild et al., 2016). Furthermore, the extent to which rescue workers suppressed their emotional arousal during an experimental emotion regulation paradigm was predictive for subsequent intrusions (Shepherd and Wild, 2014). These findings suggest a central role of rumination and suppression in the development of trauma-related mental disorders instead of being an epiphenomenon or a consequence of the respective disorders (e.g., Michael et al., 2007; Kleim et al., 2012).

Regarding adaptive emotion regulation strategies, research obtained mixed results. Some studies report reappraisal to be linked to less severe post-traumatic symptoms (Shepherd and Wild, 2014), whereas others found no relationship with rescue workers’ mental health (Beaton et al., 1999; Clohessy and Ehlers, 1999). Moreover, to our knowledge, no study has investigated the emotion regulation strategies acceptance and problem solving in rescue workers. Finally, no study so far investigated the association of emotion regulation with perceived work-related stress. Given that the perception of chronic work-related stress represents a central risk factor for the development of health impairments (e.g., Aasa et al., 2005), it is of great interest to investigate whether and to what extent emotion regulation strategies are associated with perceived work-related stress in rescue workers.

In sum, past research has shown associations between several emotion regulation strategies and perceived work-related stress and stress-related symptoms in healthy and clinical populations. However, little is known about whether and how rescue workers use specific emotion regulation strategies in their daily duties and how this relates to their physical and mental health. Therefore, the *first aim* of this cross-sectional study was to examine to what extent rescue workers use the six commonly studied emotion regulation strategies acceptance, reappraisal, problem solving, avoidance, suppression, and rumination. Our *second aim* was to investigate the relationship between these strategies and the rescue workers’ perceived work-related stress and mental health. In detail, we hypothesized that adaptive emotion regulation strategies (i.e., acceptance, problem solving, reappraisal) are linked to reduced work-related stress and fewer post-traumatic, depressive, and somatic symptoms. On the contrary, we expected that maladaptive emotion regulation strategies (i.e., rumination, suppression, avoidance) are associated with the opposite effects. Independent of emotion regulation, we expected general workload and recent private or work-related stressful events to elevate the perception of work-related stress. Further, we expected the number of potentially traumatic life events and increased work-related stress to predict more severe post-traumatic, depressive, and somatic symptoms. Therefore, we controlled for these potential covariates and were especially interested in incremental effects of emotion regulation strategies beyond these factors.

## Materials and Methods

### Participants and Procedure

All participants were employees of two German Red Cross emergency medical service stations. During occupational health seminars, 318 rescue workers were introduced to the study’s aims and procedure and received an individual link for participation in an online survey designed to assess stress and symptom burden and to examine possible resilience and vulnerability factors. Of 115 rescue workers participating in the survey, full data was available from 103 participants. Data of one participant had to be excluded due to an invariant response pattern, leaving *N* = 102 participants (32.1% of the regional working population) as the final sample, including 66 men and 36 women. The survey was presented in LimeSurvey (LimeSurvey GmbH, 2017) and took about 1 h to complete. All subjects gave written informed consent in accordance with the Declaration of Helsinki. The protocol was approved by the Ulm University Ethics Committee. There was no compensation for participation. On request, participants received individual feedback on their results.

In terms of formation, the sample comprised 30 (29.4%) emergency medical technician intermediates (EMT-I/85, Ger.: *Rettungssanitäter*), 61 (59.8%) EMT paramedics (EMT-P; Ger.: *Notfallsanitäter*) as well as 10 EMT-P trainees (9.8%) and one member of the rescue coordination center (1.0%). The study sample was representative for the total number of employees in both rescue stations (see Supplementary Table 1). Population and sample had a similar age distribution, with the population slightly older than the sample. This is possibly the result of a slight under-representation of medical student volunteers in the sample.

### Measures

#### Emotion Regulation

To comprehensively assess the use of emotion regulation strategies, we employed several commonly used inventories (cf. Aldao et al., 2010): *Acceptance* was measured using the respective four COPE items (Carver et al., 1989) in their German translation (Cronbach’s α = 0.83). *Problem solving* was assessed by combining the four items of the scales for active coping and planning taken from the German version of the BriefCOPE (Knoll et al., 2005; Cronbach’s α = 0.70). *Reappraisal* and *suppression* were assessed using the German Emotion Regulation Questionnaire (ERQ; Abler and Kessler, 2009; Cronbach’s α = 0.69 and 0.82, respectively). *Rumination* was assessed using the symptom-focused rumination scale of the German Response Styles Questionnaire (RSQ; Bürger and Kühner, 2007; Cronbach’s α = 0.81). *Avoidance* was assessed using the avoidance scale of the Coping Strategies Inventory (CSI; Tobin et al., 1984; Cronbach’s α = 0.79). To obtain comparable value dimensions, all emotion regulation scales were transformed in 0 to 10.

#### Outcome Measures

##### Perceived work-related stress

To assess rescue workers’ perceived stress due to the particular operational and organizational work factors of the emergency medical service we developed a specific questionnaire. On seven items, participants reported their stress experience due to alarms, shift work, interruptions of meals, sweating caused by heavy work clothing, or the loud sound of the emergency alarm. Participants could add another stressful work factor as free text. Responses were recorded on a four-point Likert scale, anchored at 1 (*not bothering*) and 4 (*very bothering*), or as 0 if the work factor was not experienced. Responses were aggregated to a sum score ranging from 0 to 32, showing a satisfactory internal consistency (Cronbach’s α = 0.81). A principal component factor analysis and Velicer’s revised minimum average partial test confirmed the scale as unidimensional. Questionnaire development and dimensionality analysis are detailed in the Supplementary Material.

##### Mental and somatic symptoms

Stress-related depressive symptoms were measured with the German Patient Health Questionnaire scale for depression (PHQ-9; Löwe et al., 2002). Participants reported on a four-point Likert scale ranging from 0 (*not at all*) to 3 (*almost every day*) how much they felt bothered by nine depressive symptoms (e.g., “tiredness or feeling of no energy”) during the past 2 weeks. The sum score of all items (ranging from 0 to 27) was used for subsequent statistical analyses (Cronbach’s α = 0.83). Somatic symptoms were measured with the German Patient Health Questionnaire scales for physical symptoms (PHQ-15; Löwe et al., 2002), including 13 items assessing how much participants felt bothered by physical-somatic symptoms during the last 4 weeks (e.g., stomach aches or back pain) and two additional items of the previously described PHQ-9 covering sleep disturbances within the last 2 weeks. Responses are recorded on a three-point Likert scale ranging from 0 (*not at all*) to 2 (*very strong*) and aggregated to a sum score ranging from 0 to 28 for statistical analyses (Cronbach’s α = 0.84). The item for menstrual pain was excluded to avoid a systematic gender bias. Post-traumatic symptoms were measured with the German version of the PTSD Checklist for DSM-5 (PCL-5; Ehring et al., 2014). With regard to their worst lifetime event, participants indicated on 20 items how much they were bothered by symptoms of the four PTSD symptom clusters intrusions, hyperarousal, avoidance, and negative alterations in mood or cognition during the last month. Responses were recorded on a five-point Likert scale ranging from 1 (*not at all*) to 5 (*very strong*) and aggregated to a sum score (ranging from 0 to 80) used for subsequent analyses (Cronbach’s α = 0.91).

For a better descriptive overview, we applied the questionnaires’ cutoffs to examine whether participants met the screening criteria for a potential diagnosis. In the PHQ-9, the cutoff for moderate depressive symptoms is reached when at least five of the nine items were answered with “on more than half of the days.” The PHQ-15 cutoff for mild somatic symptoms is reached when at least 3 out of 14 items were answered with “bothered a lot” and a sum score of at least six points is reached. The PCL-5 cutoff for a suspected PTSD diagnosis is reached when a sum score of at least 33 points is reached. According to screening cutoffs, *n* = 4 (3.9%) participants fulfilled criteria for a PTSD, *n* = 10 (9.8%) showed moderate levels of depressive symptoms, and *n* = 16 (15.7%) showed moderate levels of somatic symptoms.

#### Control Variables

##### Workload

To control the influence of the average quantitative workload on perceived work-related stress (cf. Heringshausen et al., 2010), rescue workers reported the number of night shifts, emergency missions (under time pressure) and routine missions (without time pressure) they completed on average per month. Since fewer personnel is present during night shifts, more emergency missions are carried out per rescue worker. In the case of part-time employment, primarily the number of night shifts is reduced and, hence, less emergency missions are carried out. Due to these interdependencies and present right-skewed distributions, the variables were split by the median (e.g., rather less or more night shifts) and combined to a workload index (minimum: 0, maximum: 3). Higher values indicated that rescue workers worked more frequently at night and performed more emergency and routine missions.

##### Current strain

Stress due to negative events during the last 4 weeks was assessed to control its potential impact on participants’ perceived stress and symptoms. Separated according to private life and work, participants indicated whether and when a stressful event occurred and briefly characterized it in a free text item. They rated the current event-related stress on a Likert scale ranging from 0 (*not stressful at all*) to 3 (*very stressful*). If no recent stress event was reported the rating scale was coded 0. The perceived stress ratings for private life and work were summarized to control for participants’ current strain in subsequent analyses.

##### Potentially traumatic life-events

Three questionnaires were employed to assess rescue workers’ exposure to potentially traumatic life-events accumulated during childhood, in private later-life, and in the emergency medical service, respectively. Childhood maltreatment was assessed using a short-version of the German Maltreatment and Abuse Chronology of Exposure scale as self-report (KERF-20; cf. Isele et al., 2014). On 20 items, participants indicated whether they had experienced different forms of emotional, physical, and sexual abuse as well as emotional and physical neglect by parents, siblings, and peers during childhood. Using the German Life Events Checklist for DSM-5 (LEC-5; Ehring et al., 2014), it was assessed whether participants had experienced or witnessed 16 potentially traumatic event types during private later-life, including the exposure of natural disasters, accidents, interpersonal violence, war, life-threatening illness or injury. An additional open item asked for other events not included in the event list. Unlike the original inventory, the response option of being confronted with events during work was excluded. Instead, work-related traumatic event exposure was assessed in more detail using the Rescue and Emergency Situations Questionnaire (RESQ). The version of Schoch (unpublished) was revised and extended by 17 items in a focus group with five experienced rescue workers (detailed information on the validation of the RESQ are given elsewhere). Participants indicated for each of the 31 different event types whether they had experienced a corresponding event during work for the emergency medical service (e.g., “Mission during which resuscitation measures remained unsuccessful”). In addition, they had the possibility to add event types that were not listed as free text. Responses were aggregated to a sum score. To represent the relative number of experienced potentially traumatic events across childhood, private later-life, and work, the centered sum scales of the three measures were averaged to a composite that served as a control variable in subsequent analyses.

### Data Analyses

Statistical analyses were performed in R Core Team (2016). Inter-correlations were analyzed using non-parametric Spearman ρ correlations because several variables were not normally distributed. Differences in the use of emotion regulation strategies were examined using Friedman’s rank sum test followed by Conover’s all-pairs *post hoc* comparison tests (using “PMCMR”; Pohlert, 2018). *p*-Values were corrected by applying the Benjamini–Hochberg false discovery rate, Cohen’s *r* served as effect size measure (Fritz et al., 2012).

The association of emotion regulation with perceived work-related stress was investigated using a multiple linear regression. Workload and current strain served as covariates. Model residuals were normally distributed and not autocorrelated, but heteroscedastic (Koenker test: *BP* = 18.32, *p* = 0.019). As a correction, the model was 5000 times Wild bootstrapped (using “hcci”; Marinho and Cribari-Neto, 2014). Further, the association of emotion regulation with post-traumatic, depressive, and somatic symptoms was examined with a multivariate linear regression. Perceived work-related stress, the number of potentially traumatic life-events, and current strain served as covariates. Checking the model assumptions indicated violated multivariate normality of model residuals (Energy test: *E* = 1.90, *p* < 0.001 using “MVN”; Korkmaz et al., 2018), particularly due to right-skewed distribution of post-traumatic and somatic symptoms. Thus, we applied M-estimators to calculate a robust multivariate regression (using “heplots”; Fox et al., 2018). Subsequent robust M-estimator based univariate regressions were performed to investigate differential associations of emotion regulation strategies with the different symptom types. Partial η^2^ served as effect size measure in regressions (Fritz et al., 2012). Our sample size enabled to detect effects of about 7.3% explained variance (η^2^) at an alpha level of 0.05 and a power of 0.80, as determined using G^∗^Power 3.1 (Faul et al., 2009) for a single predictor.

## Results

### Use of Emotion Regulation Strategies

**FIGURE 1:**
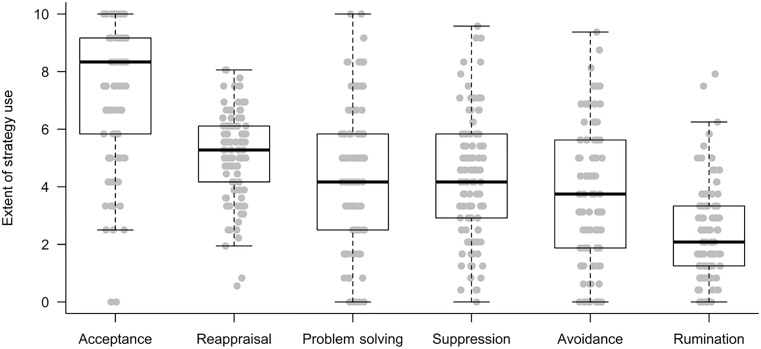
Use of emotion regulation strategies.

The use of emotion regulation strategies differed significantly, Friedman-χ^2^(5) = 166.53, *p* < 0.001 (see Figure 1 and Table 1). Acceptance and reappraisal were more frequently used than problem solving, suppression, and avoidance, which in turn were more frequently used than rumination (for *post hoc* tests see Supplementary Table 2). Maladaptive strategies were all positively correlated with each other (ρ = 0.23-0.33, *p*’s ≤ 0.021), whereas adaptive strategies were not (*p*’s ≥ 0.244). There were no correlations between the use of adaptive and maladaptive strategies, except for a positive association of rumination and problem solving (ρ = 0.22, *p* = 0.028). Supplementary Table 3 presents descriptive statistics and correlations of all study variables.

**Table 1:** Bivariate Spearman correlations of emotion regulation strategies with perceived work-related stress and stress-related symptoms (*N* = 102).

	*Mdn* (*IQR*)^†^	Perceived work-related stress	Post-traumatic symptoms	Depressive symptoms	Somatic symptoms
Acceptance	8.33 (3.33)	-0.09	-0.21^*^	-0.25^*^	-0.29^**^
Reappraisal	5.28 (2.01)	-0.05	-0.05	-0.03	-0.05
Problem solving	4.17 (3.33)	0.16	0.19	0.12	0.08
Avoidance	3.75 (3.75)	-0.01	0.23^*^	0.26^**^	0.24^*^
Suppression	4.17 (2.92)	0.24^*^	0.44^***^	0.48^***^	0.41^***^
Rumination	2.08 (2.08)	0.32^**^	0.51^***^	0.67^***^	0.60^***^


*^∗^*p* < 0.050, ^∗∗^*p* < 0.010, ^∗∗∗^*p* < 0.001, two-tailed. ^†^The sum scores of the employed emotion regulation strategy scales were transposed on a range of 0 to 10 for comparability.*

### Emotion Regulation and Perceived Work-Related Stress

Rescue workers reported moderate work-related stress (*Mdn* ± *IQR* = 14.00 ± 7.00), which was elevated in rescue workers who had a higher workload (ρ = 0.23, *p* = 0.019), and who reported current strain due to an acute private or work-related major stress event (ρ = 0.20, *p* = 0.039). Bivariate correlations (Table 1) indicated that the use of suppression and rumination was associated with higher work-related stress. Other emotion regulation strategies did not significantly correlate with work-related stress.

To examine the association of emotion regulation with perceived work-related stress while controlling for workload and current strain, a multiple linear regression was performed, *F*(8,93) = 3.78, *p* < 0.001, *R*^2^ = 24.5%. In detail, perceived work-related stress was associated with higher workload (β = 0.27, *p* = 0.003, $\eta_{p}^{2}$ = 0.086) and higher use of rumination (β = 0.18, *p* = 0.044, $\eta_{p}^{2}$ = 0.042). In contrast, avoidance was linked to less work-related stress (β = -0.19, *p* = 0.034, $\eta_{p}^{2}$ = 0.046). As a trend, suppression (β = 0.19, *p* = 0.067, $\eta_{p}^{2}$ = 0.035) and current strain (β = 0.18, *p* = 0.074, $\eta_{p}^{2}$ = 0.033) were linked to more work-related stress. There were no significant effects of other emotion regulation strategies on perceived work-related stress (see Table 2). Partial regression plots for the associations between emotion regulation strategies and perceived work-related stress are given in Supplementary Figure 1.

**Table 2:** Results of linear regression analyses (*N* = 102).

	Perceived work-related stress	Post-traumatic symptoms	Depressive symptoms	Somatic symptoms	Multivariate tests^a^
***Predictors***	**β**	**β**	**β**	**β**	**Λ**

Acceptance	-0.11	-0.13(^*^)	-0.07	-0.14^*^	0.935
Reappraisal	0.03	-0.04	-0.03	-0.02	0.994
Problem solving	0.05	0.12	-0.05	-0.03	0.962
Avoidance	-0.19^*^	0.11	0.07	0.07	0.970
Suppression	0.19(^*^)	0.21^**^	0.29^***^	0.22^**^	0.780^***^
Rumination	0.18^*^	0.29^**^	0.42^***^	0.31^***^	0.690^***^
***Covariates***					
Workload	0.27^**^	-	-	-	-
Perceived work-related stress	-	0.08	0.20^**^	0.22^**^	0.879^**^
Current strain	0.18(^*^)	0.03	-0.03	-0.06	0.989
Potentially traumatic life-events	-	0.15(^*^)	0.16^*^	0.14(^*^)	0.910^*^
*R*^2^	24.5%	45.4%	67.7%	49.4%	-
*F*(df_1_,df_2_)	3.78^***^	8.49^***^	21.43^***^	9.99^***^	-
	(8,93)	(9,92)	(9,92)	(9,92)	


*(^∗^)p < 0.075, ^∗^*p* < 0.050, ^∗∗^*p* < 0.010, ^∗∗∗^*p* < 0.001, two-tailed. Standardized regression coefficient (β). ^*a*^The association of predictors and covariates with the overall severity of post-traumatic, depressive, and somatic symptoms was tested using multivariate tests, Wilk’s Lambda (Λ). Detailed results are presented in the Supplementary Tables 4-6.*

### Emotion Regulation and Post-traumatic, Depressive, and Somatic Symptoms

The sample showed rather mild to moderate post-traumatic (*Mdn* ± *IQR* = 6.5 ± 12.0), depressive (*Mdn* ± *IQR* = 3.0 ± 6.3), and somatic symptoms (*Mdn* ± *IQR* = 5.0 ± 6.0), with a small number of participants with more severe symptoms (right-skewed distribution). The number of experienced potentially traumatic life events (ρ = 0.39-0.43, *p*’s < 0.001) and perceived work-related stress (ρ = 0.28-0.44, *p*’s ≤ 0.005) were associated with increased symptom severity. In addition, current strain was linked to more severe post-traumatic and depressive symptoms (both ρ = 0.21, *p* = 0.034). Regarding emotion regulation, bivariate correlations (Table 1) indicated that avoidance, suppression, and rumination were linked to generally more severe symptoms, whereas acceptance was associated with less severe symptoms. Reappraisal and problem solving did not significantly correlate with symptom severity.

A robust multivariate regression analysis was performed to investigate the association of emotion regulation strategies and overall symptom severity (i.e., post-traumatic, depressive, and somatic symptoms were combined as a multivariate outcome). Perceived work-related stress, the number of potentially traumatic life-events, and current strain served as covariates. Robust multivariate tests confirmed that rescue workers’ overall symptom severity was linked to the more frequent use of rumination (Wilk’s Λ = 0.690, *p* < 0.001, $\eta_{p}^{2}$ = 0.310) and suppression (Wilk’s Λ = 0.780, *p* < 0.001, $\eta_{p}^{2}$ = 0.220). In addition, symptom severity was higher in individuals who perceived more work-related stress and who were exposed to more potentially traumatic life-events (see Table 2).

Subsequent robust univariate linear regressions were computed to analyze the relationship of emotion regulation with post-traumatic, depressive, and somatic symptoms as separate outcomes (Table 2). Overall, the models explained *R*^2^ = 45–67% of variance. Post-traumatic symptoms were increased in rescue workers who more frequently used rumination (β = 0.29, *p* = 0.002, $\eta_{p}^{2}$ = 0.101) and suppression (β = 0.21, *p* = 0.010, $\eta_{p}^{2}$ = 0.070) and, as a trend, who had experienced more potentially traumatic life-events (β = 0.15, *p* = 0.057, $\eta_{p}^{2}$ = 0.039). As a trend, fewer symptoms occurred in individuals who more often used acceptance (β = -0.13, *p* = 0.070, $\eta_{p}^{2}$ = 0.035). There were no significant effects of other emotion regulation strategies, perceived stress or current strain on post-traumatic symptom severity (see Table 2).

Moreover, rumination (β = 0.42, *p* < 0.001, $\eta_{p}^{2}$ = 0.283) and suppression (β = 0.29, *p* < 0.001, $\eta_{p}^{2}$ = 0.197) were more frequently used by rescue workers who reported more severe depressive symptoms. The two strategies accounted for about half of the variance of the sample’s depressive symptoms. In addition, the number of experienced potentially traumatic life-events (β = 0.16, *p* = 0.011, $\eta_{p}^{2}$ = 0.068) and perceived work-related stress (β = 0.20, *p* = 0.002, $\eta_{p}^{2}$ = 0.104) were associated with more depressive symptoms. No effects on depressive symptoms were observed for other emotion regulation strategies and current strain (see Table 2).

Rumination (β = 0.31, *p* = 0.001, $\eta_{p}^{2}$ = 0.122) and suppression (β = 0.22, *p* = 0.004, $\eta_{p}^{2}$ = 0.087) as well as perceived work-related stress (β = 0.22, *p* = 0.004, $\eta_{p}^{2}$ = 0.087) and, as a trend, potentially traumatic life-events (β = 0.14, *p* = 0.064, $\eta_{p}^{2}$ = 0.037) were linked to more severe somatic symptoms. Conversely, the use of acceptance went along with less somatic symptoms (β = -0.14, *p* = 0.046, $\eta_{p}^{2}$ = 0.042). None of the other emotion regulation strategies or covariates had significant effects on somatic symptoms (see Table 2). Partial regression plots for the associations between emotion regulation strategies and stress-related symptoms are given in Supplementary Figure 1.

## Discussion

This study investigated the use and relationship of six emotion regulation strategies on perceived work-related stress and stress-related depressive, post-traumatic, and somatic symptoms in rescue workers. Our results showed that rescue workers generally use more adaptive than maladaptive emotion regulation strategies. Furthermore, rumination and suppression were associated with more work-related stress and more post-traumatic, depressive, and somatic symptoms. Surprisingly, avoidance predicted less work-related stress in a regression analysis, but correlated with more severe symptoms. On the contrary, acceptance correlated with lower symptom severity. The adaptive emotion regulation strategies reappraisal and problem solving showed no significant associations with work-related stress and stress-related symptoms. This could indicate that different populations, depending on their respective requirements, develop specific regulatory styles that do not follow the classical distinction between adaptive and maladaptive strategies.

### Use of Emotion Regulation Strategies

Descriptively, rescue workers used maladaptive emotion regulation strategies to a comparable or even slightly less extent than people in the community samples. However, they used adaptive emotion regulation strategies more frequently than maladaptive strategies and comparably more frequently than people in the community samples (for more details, see Supplementary Table 7).

### The Relationship of Emotion Regulation and Perceived Work-Related Stress and Stress-Related Symptoms

#### Maladaptive Emotion Regulation

Regarding maladaptive emotion regulation strategies, previous studies in community samples and emergency medical personnel indicated stable associations between the use of rumination, suppression, and avoidance with a substantially increased risk for and severity of post-traumatic and depressive symptoms (Beaton et al., 1999; Clohessy and Ehlers, 1999; Michael et al., 2007; Aldao et al., 2010; Kirby et al., 2011; Webb et al., 2012; Razik et al., 2013; Ehring and Ehlers, 2014; Shepherd and Wild, 2014; Wild et al., 2016). In line with these findings, rescue workers’ use of rumination, suppression, and avoidance correlated with more severe post-traumatic and depressive symptoms in this study. The effect sizes were the highest found in this study and comparable to the meta-analytical effect sizes reported by Aldao et al. (2010). Importantly, we observed a comparable relationship for somatic symptoms as well, indicating that using maladaptive emotion regulation strategies could impair rescue workers’ health more broadly than previously reported (cf. Hegg-Deloye et al., 2014).

Multiple linear regressions confirmed the robustness of these relationships for rumination and suppression under control of other emotion regulation strategies as well as relevant trauma and stress variables, whereas avoidance demonstrated no significant effect. The fact that avoidance showed no incremental value beyond suppression and rumination could be explained by the strategies’ relationship: bivariate correlations indicated that the use of both avoidance and suppression were associated with an increased use of rumination. Thus, the strategies likely explain shared variance in the regression analysis. A possible explanation why suppression and rumination (instead of avoidance) remain significant could be derived from the literature: there is evidence that suppression of negative emotions and thoughts provokes a “rebound effect” (also called paradoxical effect of thought suppression), that is, subsequently, the suppressed memories and emotions intrude consciousness relentlessly (e.g., Wenzlaff and Wegner, 2000; Dalgleish et al., 2008). Indeed, in traumatized rescue workers, suppression was associated with more intrusions in the long term (Shepherd and Wild, 2014). As a result, suppression can lead to increased rumination and, thereby, accelerate the development of post-traumatic, depressive, and physical symptoms in the long term (e.g., Moore et al., 2008; Dickson et al., 2012).

Moreover, it is important that rumination and suppression were also linked to an elevated perception of work-related stress. The observed effect sizes were smaller than for the stress-related symptoms. If negative emotions are regulated in a maladaptive way, even more negative emotions may arise, leading to increased post-traumatic, depressive, and physical symptoms. Regarding their dysfunctional mechanisms, rumination and suppression may prevent emotional processing of emotionally distressing or even traumatic events (Rachman, 1990; Clohessy and Ehlers, 1999). By permanently thinking about the negative events of the past, a sense of current threat may maintain in form of rumination (Ehlers and Clark, 2000). Longitudinal studies with emergency medical service trainees (Wild et al., 2016) and traumatized adults (e.g., Michael et al., 2007; Kleim et al., 2012) indicated the tendency to ruminate about traumatic events as a prospective predictor for PTSD. Notably, we investigated rumination as a tendency to ruminate about past events in general, as opposed to previous studies (e.g., Clohessy and Ehlers, 1999; Razik et al., 2013; Wild et al., 2016) that focused on trauma-related rumination. Therefore, our results highlight the adverse role of rumination *per se* (cf. Michael et al., 2007).

In contrast to other studies (Aldao et al., 2010; Razik et al., 2013), avoidance was associated with less work-related stress, indicating unexpected beneficial effects of this strategy. Indeed, to overcome highly emotional disturbing events, strategies like avoidance or distraction are preferred as they provide an effective short-term reduction of negative feelings (Gross, 1998; Webb et al., 2012; Schönfelder et al., 2014; Levy-Gigi et al., 2015). At the same time, avoidance was positively correlated with the use of ruminaton as well as more severe post-traumatic, depressive, and physical symptoms, which in turn is in line with studies showing negative long-term effects of avoidance on the mental and physical health of rescue workers (Beaton et al., 1999; Clohessy and Ehlers, 1999; Kirby et al., 2011; Razik et al., 2013; cf. the review by Aldao et al., 2010). In sum, our results support the view that avoidance is associated with less perceived work-related stress in the short term, but likely at the cost of more rumination and more severe mental and physical health promblems in the long term.

#### Adaptive Emotion Regulation

Regarding adaptive emotion regulation strategies, reappraisal and problem solving were no substantial predictors of work-related stress and stress-related symptoms. This contradicts previous meta-analytical findings (Prati and Pietrantoni, 2009; Aldao et al., 2010; Webb et al., 2012), where reappraisal and problem solving produced medium-sized effects in preventing health impairments and stimulating post-traumatic growth in clinical and community samples. One explanation could be that “adaptive” strategies might not always be beneficial in all contexts (Bonanno and Burton, 2013; Aldao et al., 2015). An integral part of the occupational work of rescue workers is the constant exposure to emotionally challenging or adverse mission events. Given that, a problem-solving approach concerning these experiences might not be applicable (e.g., in case of death, natural disasters, or criminality) or might even imply personal retreat (e.g., resignation from job in order to avoid such experiences). Intriguingly, problem solving correlated positively with rumination. A reason for this counterintuitive finding might be that people often engage in rumination by persistently trying to understand and solve their problems (Papageorgiou and Wells, 2003; Ayduk and Kross, 2010). One might speculate that in our sample, rescue workers who used problem solving also engaged in rumination more often since they “confused” their attempts to solve a problem with repetitively brooding about unchangeable negative mission experiences without finding a satisfying solution. Similarly, reappraisal might be an ineffective strategy in this context, since certain operational events cannot be re-interpreted as events with a positive meaning if they are the arbitrary product of tragic coincidence (e.g., sudden infant death). In fact, our findings are in line with previous studies in rescue workers indicating no beneficial effect of positive reinterpretations of experienced adverse events on mental health (Beaton et al., 1999; Clohessy and Ehlers, 1999; but see Shepherd and Wild, 2014).

In contrast to reappraisal and problem solving, acceptance was associated with less severe post-traumatic, depressive, and somatic symptoms. In the multiple regression analyses, acceptance was also linked to reduced perception of work-related stress. In our study, acceptance was in fact the only adaptive emotion regulation strategy that showed beneficial effects on perceived work-related stress and mental and physical health of rescue workers. The associations were only small to moderate in size, but fit to the findings of a meta-analysis by Prati and Pietrantoni (2009). Increasingly popular therapy approaches involve the use of acceptance and related mindfulness techniques guiding patients to adopt an observing, non-judgmental (i.e., accepting) relationship to their feelings, thoughts, and beliefs (e.g., *Acceptance and Commitment Therapy*, Hayes et al., 2006; *Dialectical Behavior Therapy*, Robins et al., 2001; *Mindfulness Based Cognitive Therapy*, Segal et al., 2002). These therapeutic techniques allow, above all, the development of psychological flexibility, which leads to a flexible adaptation of behavior in difficult life situations considering personal values and the type of experience (Hayes et al., 2006). On the other hand, the non-acceptance of stressful personal experiences is associated with the constant attempt to find a solution or to seek a different interpretation of what happened. This leads to greater occupation by these events and thereby an increase in rumination and, as a result, more emotional suffering. Through the acceptance of what happened, a self-distancing perspective is adopted when trying to analyze thoughts and feelings as past events, which in turn can prevent the development of negative counterproductive emotions and therefore the initiation of a rumination process (Papageorgiou and Wells, 2003; Ayduk and Kross, 2010). There is evidence that acceptance of psychological distress or physical pain reduces the tendency to ruminate over these experiences (Lakhan and Schofield, 2013). Acceptance and psychological flexibility are associated in turn with greater mental health (e.g., Kashdan and Rottenberg, 2010). In sum, our results indicate that acceptance is a beneficial strategy to cope with the consequences of work-related stress and goes along with less work-related stress as well as mental and physical symptoms, although the effect size seems to be rather small.

The general picture emerging from our study is that rescue workers use more adaptive than maladaptive emotion regulation strategies. Of these strategies, however, only acceptance was associated with a beneficial outcome (i.e., reduced work-related stress and less severe stress-related symptoms). In contrast, already the moderate use of maladaptive strategies was associated with detrimental effects on perceived work-related stress and stress-related symptoms. Thus, although rescue workers in our study used reappraisal, problem solving and acceptance rather frequently, the use of maladaptive emotion regulation strategies seems to exert more deleterious effects that cannot be prevented by the use of adaptive strategies. If replicated in further research, this may have important clinical implications. First, suppression and rumination have been identified as stable predictors of perceived work-related stress and stress-related symptoms in rescue workers. Therefore, targeting both strategies may be helpful for the prevention of occupational health impairments in trauma-exposed professions. Second, our findings suggest that a reduction of maladaptive emotion regulation strategies may be more promising than the promotion of adaptive emotion regulation strategies alone. Third, the use of reappraisal and problem solving might even facilitate the initiation of rumination processes and should therefore be carefully evaluated in prevention and intervention approaches. Instead, future research could investigate whether acceptance-based trainings could contribute to maintain rescue workers’ mental and physical health. Most importantly, the current study points toward the necessity to further investigate the processes that can explain *why* rescue workers engage in maladaptive emotion regulation strategies despite their negative long-term consequences. For example, it would be helpful to investigate the particular short- and long-term intentions of rescue workers in applying or non-applying certain strategies in response to different types of stressors (i.e., unchangeable critical mission incidents vs. changeable working conditions) and to examine in more detail why some emotion regulation strategies might have beneficial effects in the short term but negative consequences in the long term (e.g., Levy-Gigi et al., 2015).

### Limitations and Future Directions

The study is limited by its cross-sectional design, the reliance on self-report measurements and its related vulnerability to bias. First, regarding the non-response bias, unknown differences may exist between rescue workers who chose to respond and those who did not. Especially rescue workers who did not participate in the study because they were currently or chronically ill or left the profession prematurely by dismissal or early retirement would have been particularly interesting regarding the consequences of stress and ineffective emotion regulation styles. Therefore, these missing cases may have led to an underestimation of effects (cf. *healthy worker effect*, Costa, 2003). However, the response rate in our sample was higher than in past research on similar occupational groups (Skeffington et al., 2017). Further, prevalence rates of mental disorders assessed by self-report questionnaires and standardized interviews are comparable to other studies in the emergency medical service (Donnelly, 2012) and lie in a normal range of Western samples (cf. general symptom prevalence in emergency service ranging from 6% to more than 20%; Martin et al., 2009). Second, survey research is vulnerable to social desirability bias (although a questionnaire assessing social desirability indicated no such behavior for any participant). This may be especially problematic in rescue workers since the expression of distress and consequences of traumatic stress have to be concealed often (Halpern et al., 2009). Therefore, the generalizability of the findings and the rate of reported health problems or psychopathological symptoms may be reduced. Third, by using a cross-sectional study design the results can only be interpreted correlational. Longitudinal studies are necessary to investigate the particular mechanisms and causal directions behind the observed associations between the use of emotion regulation strategies, psychopathological symptoms and exposure to potentially traumatic life events and work-related stress. In addition, the sample size of the current study was comparatively small for correlation and regression analyses (Schönbrodt and Perugini, 2013). Although our sample size enabled us to detect effects of about 7.3% variance explanation, the investigation of smaller effects and complex interaction effects between emotion regulation and physical and mental health requires larger samples with more statistical power.

## Conclusion

In sum, our results demonstrate that rescue workers generally use more adaptive than maladaptive emotion regulation strategies. Although reappraisal and problem solving were associated with beneficial health outcomes in previous studies in healthy and clinical populations, they might be less effective in the context of the emergency medical service. In contrast, acceptance seems to be an adaptive strategy for rescue workers to deal with highly stressful and aversive events. In line with previous research, we found considerable negative effects of the maladaptive strategies rumination and suppression on perceived work-related stress and stress-related symptoms. Importantly, our results show negative consequences of these strategies not only on depressive and post-traumatic but also on somatic symptoms. Furthermore, we investigated the association of the regulation of emotions with work-related stress perception as a crucial risk factor for the development of psychopathological symptoms. Here, avoidance was associated with less stress in the short term, but likely at the cost of more severe mental and psychial health promblems in the long term. The data suggest that a reduction of rumination and suppression strategies should be targeted in prevention and intervention measures in rescue workers. Our results point toward investigating the effects of acceptance-based preventive trainings in rescue workers. Further studies are needed to replicate our findings in larger samples. Moreover, longitudinal designs and data on underlying short- and long-term motives and biological processes may provide valuable insights into causal mechanisms behind these associations.

## Data Availability statement

Restrictions apply to the datasets: the dataset of this manuscript is not publicly available because the data may not be passed on or published to third parties outside the research project. We do not have the consent of the ethics committee or the participants to grant any third parties access to or insight in all or parts of the collected data.

## Author Contributions

AG, AB, RR, DC, and I-TK developed the study concept. AB and DC conducted the study setup and data collection. AB performed the statistical data analysis. AG and AB drafted the manuscript under supervision of I-TK and RR. All authors contributed to the interpretation of data, critically revised the manuscript, and approved the final version of the manuscript for submission.

## Conflict of Interest Statement

The authors declare that the research was conducted in the absence of any commercial or financial relationships that could be construed as a potential conflict of interest.
