# Supplementary material for: Emotion Regulation in Rescue Workers: Differential Relationship With Perceived Work-Related Stress and Stress-Related Symptoms
Source: Front Psychol. 2019 Jan 10;9:2744. doi: 10.3389/fpsyg.2018.02744 (PMC6335291; doi:10.3389/fpsyg.2018.02744)
Supplement: Supplementary file 1 [file Table_1.DOCX]

Supplementary Material

Emotion regulation in rescue workers: Differential impact on perceived work-related stress and stress-related symptoms

**Anne Gärtner*, Alexander Behnke*, Daniela Conrad, Iris-Tatjana Kolassa, Roberto Rojas**

* joint first authorship

*** Correspondence:**

| Alexander Behnke  Clinical and Biological Psychology  Institute of Psychology and Education  Ulm University  89081 Ulm, Germany  Phone: +49-731-50-26591  Fax: +49-731-50-26599  Email: alexander.behnke@uni-ulm.de | Anne Gärtner  Differential and Personality Psychology  Faculty of Psychology  Technische Universität Dresden  01062 Dresden, Germany  Phone: +49-351-463-36997  Fax: +49-351-463-36993  Email: anne_gaertner@tu-dresden.de |
| --- | --- |

# Supplementary Figures and Tables

## Supplementary Tables

Supplementary **Table 1**. Population and sample characteristics

|  | Population | | Sample | |  |
| --- | --- | --- | --- | --- | --- |
| Rescue workers | absolute | relative | absolute | relative | Test statistic |
| total | 318 | 100.0% | 102 | 32.1%^#^ |  |
| by ambulance station |  |  |  |  | *χ*²(1, 420) = 0.00,  *p* = 1.000,  *V* = .00 |
| Ulm | 223 | 70.1% | 72 | 70.6% |  |
| Heidenheim | 95 | 29.9% | 30 | 29.4% |  |
| by employment |  |  |  |  | *χ*²(2, 420) = 11.84,  *p* = .003,  *V* = .17 |
| salaried | 198 | 62.3% | 77 | 75.5% |  |
| voluntary | 101 | 31.8% | 15 | 14.7% |  |
| in apprentice | 19 | 6.0% | 10 | 9.8% |  |
| by sex |  |  |  |  | *χ*²(1, 420) = 0.71,  *p* = .399,  *V* = .05 |
| men | 222 | 69.8% | 66 | 64.7% |  |
| women | 96 | 30.2% | 36 | 35.3% |  |
|  | *M* (*SD*) | *Mdn* | *M* (*SD*) | *Mdn* | Test statistic |
| Age in years | 32.1 (11.2) | 27.5 | 30.1 (11.0) | 26.0 | *z* = -2.53, *p* = .011,  *r* = -.12 |
| Time working for emergency service in months | 68.0 (66.3) | 45.0 | 92.2 (105.4) | 39.5 | *z* = -0.67, *p* = .506,  *r* = -.03 |

*Note*. *^#^* proportion of population, *n* = 318. Population and sample frequency distributions were compared using χ² tests applying a Yates correction in case of 2⨯2 tables. Age and affiliation were compared using Mann-Whitney *U*-tests because the distributions violated normality and variance equity. Effect sizes are indicated by Cramer’s contingency coefficient *V* and Cohen’s *r*.

**Supplementary Table 2**. Results of Conover’s post-hoc tests pairwise comparing the use of emotion regulation strategies (*N* = 102)

|  |  | Acceptance | Reappraisal | Problem-solving | Suppression | Avoidance | Rumination |
| --- | --- | --- | --- | --- | --- | --- | --- |
| Test statistic *t* | Acceptance | − | -4.24^***^ | -6.90^***^ | -6.22^***^ | -7.88^***^ | -12.31^***^ |
|  | Reappraisal |  | − | -2.66^*^ | -1.98 | -3.64^***^ | -8.07^***^ |
|  | Problem-solving |  |  | − | 0.68 | -0.98 | -5.41^***^ |
|  | Suppression |  |  |  | − | -1.66 | -6.09^***^ |
|  | Avoidance |  |  |  |  | − | -4.43^***^ |
|  | Rumination |  |  |  |  |  | − |
| Cohen’s *r* | Acceptance | − | -0.63 | -0.69 | -0.69 | -0.71 | -0.84 |
|  | Reappraisal |  | − | -0.26 | -0.31 | -0.45 | -0.76 |
|  | Problem-solving |  |  | − | -0.02 | -0.15 | -0.63 |
|  | Suppression |  |  |  | − | -0.18 | -0.70 |
|  | Avoidance |  |  |  |  | − | -0.50 |
|  | Rumination |  |  |  |  |  | − |
|  | *Mdn* | 8.33 | 5.28 | 4.17 | 4.17 | 3.75 | 2.08 |
|  | *IQR* | 3.33 | 1.94 | 3.33 | 2.92 | 3.75 | 2.08 |

*Note*: ^*^ *p* < .05, ^**^ *p* < .01, ^***^ *p* < .001, two-tailed, false detection rate-corrected. Cohen’s *r* indicates the effect sizes of the difference between columns and lines.

Supplementary Table 3. Descriptive statistics and Spearman’s ρ correlation-matrix of study variables (*N* = 102)

|  |  |  |  |  |  |  |  |  |  |  |  |  |  |  |  |  |
| --- | --- | --- | --- | --- | --- | --- | --- | --- | --- | --- | --- | --- | --- | --- | --- | --- |
| 1. Posttraumatic symptoms (PCL-5) | − | .59^***^ | .55^***^ | -.21^*^ | -.05 | .19 | .23^*^ | .44^***^ | .51^***^ | .06 | .28^**^ | .21^*^ | .39^***^ | .00 | .11 | -.22^*^ |
| 1. Depressive symptoms (PHQ-9) | .59^***^ | − | .82^***^ | -.25^*^ | -.03 | .12 | .26^**^ | .48^***^ | .67^***^ | .15 | .44^***^ | .21^*^ | .43^***^ | .13 | .15 | -.06 |
| 1. Somatoform symptoms (PHQ-15) | .55^***^ | .82^***^ | − | -.29^**^ | -.05 | .08 | .24^*^ | .41^***^ | .60^***^ | .15 | .41^***^ | .11 | .42^***^ | .11 | .15 | -.19 |
| 1. Acceptance (COPE) | -.21^*^ | -.25^*^ | -.29^**^ | − | -.02 | .05 | -.18 | -.08 | -.19 | .15 | -.09 | -.02 | -.09 | -.03 | .03 | .09 |
| 1. Reappraisal (ERQ) | -.05 | -.03 | -.05 | -.02 | − | .12 | .16 | .02 | .02 | -.10 | -.05 | -.10 | .03 | -.20^*^ | -.15 | .08 |
| 1. Problem-solving (BriefCOPE) | .19 | .12 | .08 | .05 | .12 | − | -.12 | .07 | .22^*^ | .06 | .16 | .22^*^ | .11 | .12 | .02 | .12 |
| 1. Avoidance (CSI) | .23^*^ | .26^**^ | .24^*^ | -.18 | .16 | -.12 | − | .23^*^ | .31^**^ | .08 | -.01 | .01 | .15 | -.10 | -.05 | -.12 |
| 1. Suppression (ERQ) | .44^***^ | .48^***^ | .41^***^ | -.08 | .02 | .07 | .23^*^ | − | .33^***^ | -.03 | .24^*^ | .02 | .25^*^ | .20 | .15 | .18 |
| 1. Rumination (RSQ-D) | .51^***^ | .67^***^ | .60^***^ | -.19 | .02 | .22^*^ | .31^**^ | .33^***^ | − | .07 | .32^**^ | .30^**^ | .29^**^ | -.09 | -.01 | -.12 |
| 1. Workload | .06 | .15 | .15 | .15 | -.10 | .06 | .08 | -.03 | .07 | − | .23^*^ | .11 | .11 | .01 | .00 | -.03 |
| 1. Perceived work-related stress | .28^**^ | .44^***^ | .41^***^ | -.09 | -.05 | .16 | -.01 | .24^*^ | .32^**^ | .23^*^ | − | .20^*^ | .22^*^ | .28^**^ | .32^**^ | .15 |
| 1. Current strain | .21^*^ | .21^*^ | .11 | -.02 | -.10 | .22 | .01 | .02 | .30^**^ | .11 | .20^*^ | − | .18 | .00 | .12 | .03 |
| 1. Potentially traumatic life-events | .39^***^ | .43^***^ | .42^***^ | -.09 | .03 | .11 | .15 | .25^*^ | .29^**^ | .11 | .22^*^ | .18 | − | .29^**^ | .34^***^ | .06 |
| 1. Age (in years) | .00 | .13 | .11 | -.03 | -.20^*^ | .12 | -.10 | .20^*^ | -.09 | .01 | .28^**^ | .00 | .29^**^ | − | .74^***^ | .46^***^ |
| 1. Time employed in emergency medical service (in years) | .11 | .15 | .15 | .03 | -.15 | .02 | -.05 | .15 | -.01 | .00 | .32^**^ | .12 | .34^***^ | .74^***^ | − | .30^**^ |
| 1. Sex^#^ | -.22^*^ | -.06 | -.19 | .09 | .08 | .12 | -.12 | .18 | -.12 | -.03 | .15 | .03 | .06 | .46^***^ | .30^**^ | − |
| Mean | 9.92 | 4.61 | 5.48 | 7.30 | 5.08 | 4.35 | 3.75 | 4.30 | 2.31 | 1.75 | 14.77 | 0.74 | 0.00 | 30.06 | 7.69 | − |
| Standard deviation | 9.75 | 4.28 | 4.43 | 2.40 | 1.52 | 2.45 | 2.41 | 2.14 | 1.64 | 1.11 | 4.76 | 1.14 | 12.40 | 11.04 | 8.79 | − |
| Median | 6.50 | 3.00 | 5.00 | 8.33 | 5.28 | 4.17 | 3.75 | 4.17 | 2.08 | 2.00 | 14.00 | 0.00 | -2.10 | 26.00 | 3.29 | − |
| Interquartile range | 12.00 | 6.25 | 6.00 | 3.33 | 2.01 | 3.33 | 3.75 | 2.92 | 2.08 | 2.00 | 7.00 | 2.00 | 13.24 | 17.00 | 10.94 | − |
| Empirical range | 0−39 | 0−18 | 0−19 | 0−10 | 0.6−8.1 | 0−10 | 0−9.4 | 0−9.6 | 0−7.9 | 0−3 | 7−27 | 0−4 | -27.4−48.6 | 18−61 | 0.1−35 | − |
| Scale range | 0−80 | 0−27 | 0−28 | 0−10 | 0−10 | 0−10 | 0−10 | 0−10 | 0−10 | 0−3 | 0−32 | 0−8 | − | − | − | − |
| Skew | 1.17 | 1.25 | 1.01 | -0.86 | -0.50 | 0.14 | 0.19 | 0.21 | 0.97 | -0.28 | 0.59 | 1.27 | 0.89 | 0.99 | 1.46 | − |
| Kurtosis | 0.49 | 1.32 | 0.47 | 0.22 | 0.19 | -0.48 | -0.94 | -0.39 | 1.08 | -1.27 | -0.40 | 0.27 | 1.85 | 0.01 | 1.22 | − |

*Note.* ^*^ *p* < .05, ^**^ *p* < .01, ^***^ *p* < .001. ^#^ Positive values indicate higher values for men (*n* = 66).

**Supplementary Table 4.** Ordinary linear regression results (*N* = 102)

| Outcome | Predictors | *b* | *SE* | *t* | *p* | η²_p_ |
| --- | --- | --- | --- | --- | --- | --- |
| Perceived work-related stress | Intercept | 11.08 | 1.88 | 5.88 | <.001^***^ | .271 |
|  | Acceptance | -0.21 | 0.15 | -1.44 | .150 | .022 |
|  | Reappraisal | 0.11 | 0.30 | 0.35 | .741 | .001 |
|  | Problem-solving | 0.10 | 0.20 | 0.52 | .614 | .003 |
|  | Avoidance | -0.37 | 0.17 | -2.12 | .034^*^ | .046 |
|  | Suppression | 0.43 | 0.23 | 1.83 | .067^(*)^ | .035 |
|  | Rumination | 0.52 | 0.26 | 2.01 | .044^*^ | .042 |
|  | Workload | 1.18 | 0.40 | 2.95 | .003^**^ | .086 |
|  | Current strain | 0.74 | 0.41 | 1.78 | .074^(*)^ | .033 |
|  | Overall model statistics: *F*(8,93) = 3.78, *p* < .001, *R*² = .245 | | | | | |

*Note*: ^(*)^ *p* < .075, ^*^ *p* < .050, ^**^ *p* ≤ .010, ^***^ *p* ≤ .001, two-tailed. Results were 5000 times Wild bootstrapped to correct for heteroscedasticity.

**Supplementary Table 5.** Robust multivariate test results (*N* = 102)

|  | Wilk’s Λ | *F*(3,90) | *p* | $\eta_{p}^{2}$ |
| --- | --- | --- | --- | --- |
| Intercept | .968 | 0.99 | .403 | .032 |
| Acceptance | .935 | 2.09 | .107 | .065 |
| Reappraisal | .994 | 0.17 | .918 | .006 |
| Problem-solving | .962 | 1.20 | .316 | .038 |
| Avoidance | .970 | 0.94 | .427 | .030 |
| Suppression | .780 | 8.48 | <.001^***^ | .220 |
| Rumination | .690 | 13.47 | <.001^***^ | .310 |
| Perceived work-related stress | .879 | 4.12 | .009^**^ | .121 |
| Current strain | .989 | 0.32 | .810 | .011 |
| Potentially traumatic life-events | .910 | 2.98 | .036^*^ | .090 |

*Note*: ^(*)^ *p* < .075, ^*^ *p* < .050, ^**^ *p* ≤ .010, ^***^ *p* ≤ .001, two-tailed.

**Supplementary Table 6.** Results of subsequent robust linear regressions (*N* = 102)

| Outcome | Predictors | *b* | *SE* | *t* | *p* | $\eta_{p}^{2}$ |
| --- | --- | --- | --- | --- | --- | --- |
| Posttraumatic symptoms (PCL-5) | Intercept | 0.46 | 4.20 | 0.11 | .913 | .000 |
|  | Acceptance | -0.53 | 0.29 | -1.83 | .070^(*)^ | .035 |
|  | Reappraisal | -0.29 | 0.47 | -0.61 | .547 | .004 |
|  | Problem-solving | 0.46 | 0.31 | 1.51 | .136 | .024 |
|  | Avoidance | 0.45 | 0.32 | 1.40 | .166 | .021 |
|  | Suppression | 0.95 | 0.36 | 2.64 | .010^**^ | .070 |
|  | Rumination | 1.75 | 0.54 | 3.22 | .002^**^ | .101 |
|  | Perceived work-related stress | 0.16 | 0.16 | 0.96 | .342 | .010 |
|  | Current strain | 0.27 | 0.68 | 0.40 | .687 | .002 |
|  | Potentially traumatic life-events | 0.12 | 0.06 | 1.93 | .057^(*)^ | .039 |
|  | Overall model statistics: *F*(9,92) = 8.49, *p* < .001, *R*² = .454 | | | | | |
| Depressive symptoms (PHQ-9) | Intercept | -1.84 | 1.40 | -1.32 | .192 | .018 |
|  | Acceptance | -0.12 | 0.10 | -1.27 | .209 | .017 |
|  | Reappraisal | -0.08 | 0.16 | -0.48 | .632 | .003 |
|  | Problem-solving | -0.09 | 0.10 | -0.91 | .367 | .009 |
|  | Avoidance | 0.13 | 0.11 | 1.17 | .247 | .015 |
|  | Suppression | 0.57 | 0.12 | 4.75 | <.001^***^ | .197 |
|  | Rumination | 1.09 | 0.18 | 6.03 | <.001^***^ | .283 |
|  | Perceived work-related stress | 0.18 | 0.05 | 3.27 | .002^**^ | .104 |
|  | Current strain | -0.12 | 0.23 | -0.53 | .597 | .003 |
|  | Potentially traumatic life-events | 0.05 | 0.02 | 2.59 | .011^*^ | .068 |
|  | Overall model statistics: *F*(9,92) = 20.68, *p* < .001, *R*² = .677 | | | | | |
| Somatoform symptoms (PHQ-15) | Intercept | 0.32 | 1.80 | 0.18 | .860 | .000 |
|  | Acceptance | -0.25 | 0.12 | -2.02 | .046^*^ | .042 |
|  | Reappraisal | -0.06 | 0.20 | -0.27 | .786 | .001 |
|  | Problem-solving | -0.05 | 0.13 | -0.36 | .721 | .001 |
|  | Avoidance | 0.12 | 0.14 | 0.89 | .376 | .009 |
|  | Suppression | 0.46 | 0.15 | 2.97 | .004^**^ | .087 |
|  | Rumination | 0.83 | 0.23 | 3.58 | .001^***^ | .122 |
|  | Perceived work-related stress | 0.21 | 0.07 | 2.97 | .004^**^ | .087 |
|  | Current strain | -0.24 | 0.29 | -0.82 | .415 | .007 |
|  | Potentially traumatic life-events | 0.05 | 0.03 | 1.88 | .064^(*)^ | .037 |
|  | Overall model statistics: *F*(9,92) = 9.99, *p* < .001, *R*² = .494 | | | | | |

*Note*: ^(*)^ *p* < .075, ^*^ *p* < .050, ^**^ *p* ≤ .010, ^***^ *p* ≤ .001, two-tailed.

**Supplementary Table 7.** Comparing the use of emotion regulation strategies with other (community) samples

|  | Present study | | Community samples† | |
| --- | --- | --- | --- | --- |
| Emotion regulation strategy | M (SD) | N | M (SD) | N |
| Acceptance | 8.8 (2.9) | 102 | 5.5 (1.6) | 2187a |
| Reappraisal | 4.1 (0.9) | 102 | 4.4 (1.0) | 2475b |
| Problem-solving | 5.2 (2.9) | 102 | 2.6 (0.6) | 241c |
| Avoidance | 1.5 (1.0) | 102 | 2.2 (0.7) | 879d |
| Suppression | 3.6 (1.3) | 102 | 4.0 (1.2) | 2475b |
| Rumination | 1.7 (0.5) | 102 | 1.9 (0.5) | 1132e |

*Note*: ^†^selected from ^a^ Doron et al. (2014), ^b^ Wiltink et al. (2011); ^c^ Hong (2007); ^d^ Tobin et al. (1984); ^e^ Michl et al. (2013). Emotion regulation scales were *not* rescaled on 0 to 10.

## Supplementary Figures


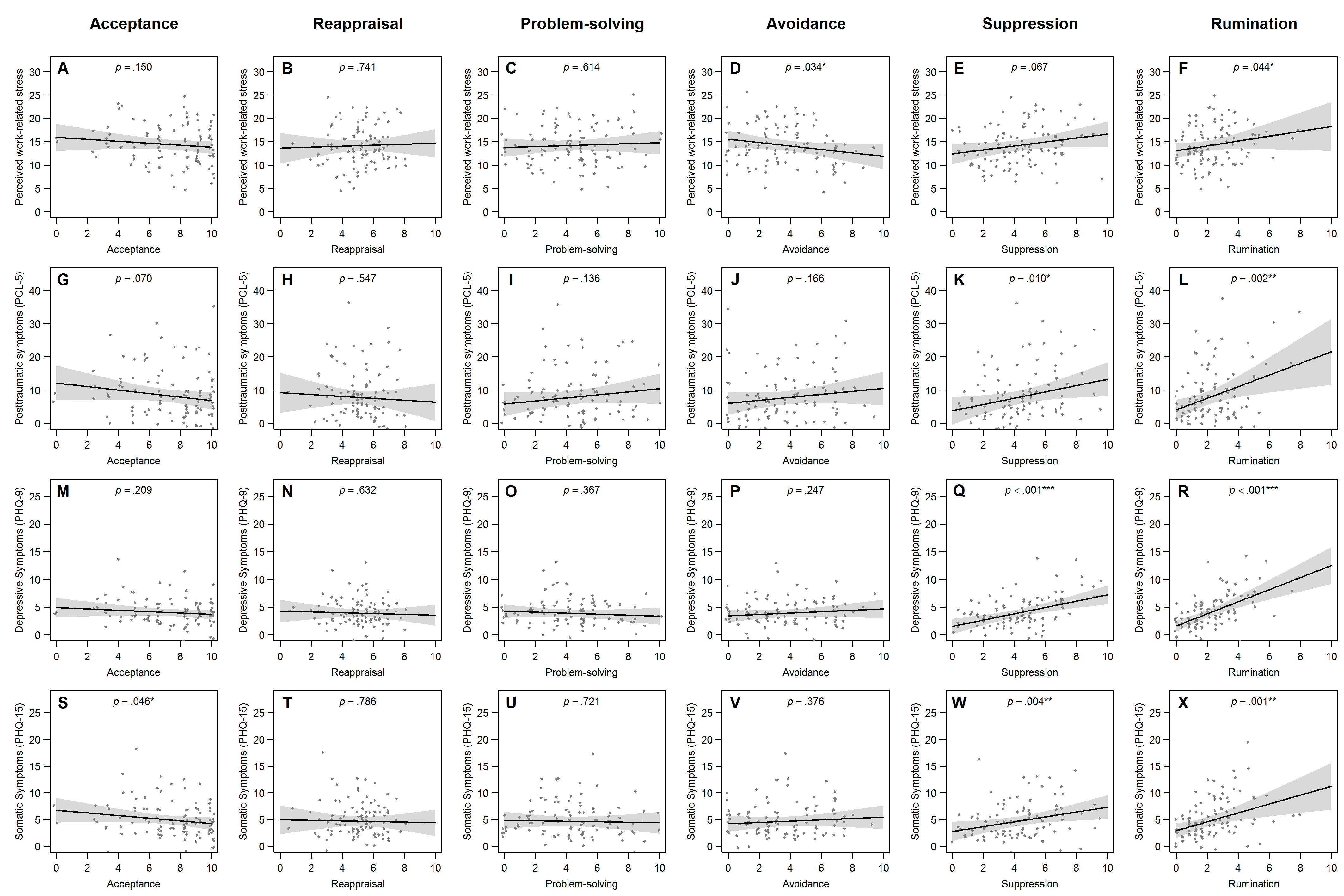


**Figure 1**. Partial regression plots for the associations of emotion regulation strategy use (sorted by column) and outcome variables (sorted by row). Influences of covariates were controlled (see Table 2 for details). Gray areas indicate 95% confidence intervals.

# Perceived work-related stress questionnaire for the emergency medical service

In a focus group with five experienced rescue workers, we developed a rating of burdensome work aspects in order to capture the specific requirements of the work requirements in the rescue service (Supplementary Table 8). Due to its bias by scale length, Cronbach’s alpha is an inconclusive measure of a scale’s homogeneity and unidimensionality (Clark & Watson, 1995). We therefore conducted a principal component exploratory factor analysis (Kaiser-Meyer-Olkin measure of sampling adequacy = .80; Bartlett’s test of sphericity, χ²(21) = 218.74, *p* < .001) and the revised Velicer’s minimum average partial (rMAP) test (O’Connor, 2000) to determine the factor structure of the scale. We found the first extracted factor to bundle 47.5% of the items’ variance. Factor loadings were sufficient, ranging from.52 to .79. The rMAP test confirmed the one-factor solution as the most appropriate because of indicating the smallest average 4^th^ power partial correlation (Supplementary Table 9).

**Supplementary Table 8.** Items of the perceived work-related stress questionnaire

|  | **German** | **English translation** |
| --- | --- | --- |
| Instruction | Im Folgenden sind Situationen beschrieben, die im Rahmen einer Tätigkeit beim Rettungsdienst erlebt und als belastend empfunden werden können. Bitte geben Sie an, ob Sie der jeweiligen Situation schon einmal ausgesetzt waren und falls ja, als wie belastend Sie diese Situationen **allgemein** empfunden haben. | The subsequent questionnaire describes situations that can be perceived as stressful in the context of working in the medical emergency service. Please indicate whether you have been exposed to the respective situation and if so, how stressful you perceived the situation **in general**. |
| Item 1 | Durch eine Alarmierung kurz vor Schichtende kommt es zu Überstunden | An alarm shortly before the end of the shift results in overtime hours. |
| Item 2 | Die Pause muss aufgrund eines plötzlichen Einsatzes unterbrochen werden, so dass Sie keine Zeit haben sich auszuruhen | The break must be interrupted due to a sudden emergency mission, so you don’t have time to rest and recover. |
| Item 3 | Das Essen muss aufgrund eines Einsatzes verschoben oder unterbrochen werden, sodass Sie sehr hungrig sind | The meal has to be postponed or interrupted due to a mission, so that you are very hungry. |
| Item 4 | Die Nachtruhe in der Nachtschicht wird durch viele Einsätze unterbrochen, sodass Sie sich übermüdet fühlen | The sleep during the night shift is interrupted by many missions, so that you feel overtired. |
| Item 5 | Ein dringender Toilettengang muss aufgrund eines Notfalleinsatzes verschoben werden | An urgent visit to the restroom must be postponed due to an emergency mission. |
| Item 6 | Erhöhtes Schwitzen durch vorgeschriebene Einsatzkleidung z.B. lange Hose oder Jacke bei Verkehrsunfall | Increased sweating due to mandatory protective clothing, e.g. long trousers or jacket in the event of a traffic accident. |
| Item 7 | Das Geräusch des Einsatzalarms, Funksprüche oder Sirenen hören | Hearing the sound of the emergency alarm, radio calls or sirens. |
| Item 8 | Irgendeine andere sehr belastende Situation:  Kurze Beschreibung: _____________________ | Some other very stressful situation:  Short description: _____________________ |

**Supplementary Table 9.** Results of exploratory factor analysis and Velicer’s revised minimum average partial test

| Extracted factor | Eigenvalue | Explained variance per factor, Δ*R*² | Cumulated explained variance, *R*² | Average partial correlation, 4th power |
| --- | --- | --- | --- | --- |
| 0 | − | − | − | 0.0339 |
| 1 | 3.32 | 47.5 | 47.5 | 0.0058 |
| 2 | .99 | 14.2 | 61.7 | 0.0353 |
| 3 | .90 | 12.8 | 74.5 | 0.0488 |
| 4 | .56 | 8.0 | 82.5 | 0.1668 |
| 5 | .51 | 7.3 | 89.7 | 0.3126 |
| 6 | .41 | 5.9 | 95.6 | 1.0000 |
| 7 | .31 | 4.4 | 100.0 | − |
